# Intake of polyphenols from cereal foods and colorectal cancer risk in the Melbourne Collaborative Cohort Study

**DOI:** 10.1002/cam4.6514

**Published:** 2023-09-13

**Authors:** Kristina Vingrys, Michael L. Mathai, Andrew J. McAinch, Julie K. Bassett, Maximilian de Courten, Lily Stojanovska, Lynne Millar, Graham G. Giles, Allison M. Hodge, Vasso Apostolopoulos

**Affiliations:** ^1^ Institute for Health and Sport Victoria University Melbourne Victoria Australia; ^2^ VU First Year College® Victoria University Melbourne Victoria Australia; ^3^ Australian Institute for Musculoskeletal Science (AIMSS) Victoria University Melbourne Victoria Australia; ^4^ Cancer Epidemiology Division Cancer Council Victoria Melbourne Victoria Australia; ^5^ Mitchell Institute for Education and Health Policy Victoria University Melbourne Victoria Australia; ^6^ Department of Nutrition and Health, College of Medicine and Health Sciences United Arab Emirates University Al Ain United Arab Emirates; ^7^ Telethon Kids Institute Nedlands WA Australia; ^8^ Centre for Epidemiology and Biostatistics, Melbourne School of Population and Global Health The University of Melbourne Parkville Victoria Australia; ^9^ Precision Medicine, School of Clinical Sciences at Monash Health Monash University Clayton Victoria Australia

**Keywords:** cereals, colorectal cancer, food frequency questionnaire, Melbourne Collaborative Cohort Study, polyphenols

## Abstract

**Background:**

Cereal‐derived polyphenols have demonstrated protective mechanisms in colorectal cancer (CRC) models; however, confirmation in human studies is lacking. Therefore, this study examined the association between cereal polyphenol intakes and CRC risk in the Melbourne Collaborative Cohort Study (MCCS), a prospective cohort study in Melbourne, Australia that recruited participants between 1990 and 1994 to investigate diet–disease relationships.

**Methods:**

Using food frequency questionnaire diet data matched to polyphenol data, dietary intakes of alkylresorcinols, phenolic acids, lignans, and total polyphenols from cereals were estimated. Hazard ratios (HRs) and 95% confidence intervals for CRC risk were estimated for quintiles of intake with the lowest quintile as the comparison category, using multivariable adjusted Cox proportional hazards models with age as the time axis adjusted for sex, socio‐economic status, alcohol consumption, fibre intake, country of birth, total energy intake, physical activity and smoking status.

**Results:**

From 35,245 eligible adults, mean (SD) age 54.7 (8.6) years, mostly female (61%) and Australian‐born (69%), there were 1394 incident cases of CRC (946 colon cancers and 448 rectal cancers). Results for total cereal polyphenol intake showed reduced HRs in Q2 (HR: 0.80; 95% CI, 0.68–0.95) and Q4 (HR: 0.75; 95% CI, 0.62–0.90), and similar for phenolic acids. Alkylresorcinol intake showed reduced HR in Q3 (HR: 0.80; 95% CI, 0.67–0.95) and Q4 (HR: 0.79; 95% CI, 0.66–0.95).

**Conclusions:**

Overall, the present study showed little evidence of association between intakes of cereal polyphenols and CRC risk. Future investigations may be useful to understand associations between cereal‐derived polyphenols and additional cancers in different populations.

## Graphical abstract

Grains and cereals may offer chemoprotection in colorectal cancer (CRC), that may partly be attributed to polyphenols from cereal foods; however, populations studies are lacking. Utilising a cereal‐specific polyphenol database designed for a specific Australian cohort, survival analysis revealed limited association between cereal polyphenol intake and CRC risk. While the study findings are in general agreement with cohort studies from total foods, further comparative studies are needed.

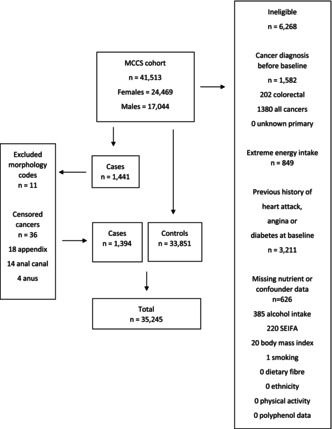

## 1 INTRODUCTION

Colorectal cancer (CRC) is a major cause of illness and mortality world‐wide. In 2020, global CRC incidence rates were estimated at 1.9 million new cases, reflecting the third most common new cancer.^1^ CRC is also the second leading cause of cancer mortality with 935,000 deaths estimated in 2020.^1^ Australia and New Zealand have some of the highest rates of colon and rectal cancer incidence, being third globally for colon and fifth for rectum.^1^ Melbourne is a multi‐ethnic major city in Australia including immigrants from southern Europe.^2^ Findings from the 30‐year Melbourne Colorectal Cancer Study suggested that first‐generation immigrants from countries with low CRC risk increased their risk to reflect Australia's higher incidence rates, likely associated with environmental factors.^3^

Dietary components are modifiable environmental factors in many cancers including CRC.^4^, ^5^ Of all cancers studied in over 80,000 US adults, cancers of the colon and rectum were estimated to have the highest number (*n* = 52,225) of new cases attributable to a sub‐optimal diet.^4^ Dietary factors associated with reduced CRC risk include the consumption of whole grains and dietary fibre;^6^ however, the association between intake of refined grains and CRC risk is less clear.^6^

While dietary fibre may contribute to CRC protection, grain and cereal foods contain bioactive polyphenols that may also contribute to chemoprotection.^7^ Polyphenols are antioxidant compounds found widely in plant foods including grain and cereal foods.^8^ Grain polyphenols largely come from non‐flavonoids, and include alkylresorcinols, phenolic acids and lignans. Polyphenols may mediate associations between grain and cereal foods and reduced CRC risk, and therefore it is important to investigate as there are specific polyphenols either uniquely or largely found in grain and cereal foods.^9^, ^10^

Experimental evidence implicates an association between cereal phenolic compounds and reduced risk of some gastrointestinal cancers.^7^, ^11^, ^12^, ^13^ For instance, alkylresorcinols in rye and wheat^10^ have shown anti‐cancer properties against colon cancer^14^, ^15^ including inhibition of HCT‐116 and HT‐29 human colon cancer cell growth,^16^ indicated via a cascade of mechanisms inducing apoptosis and cell‐cycle arrest.^14^ Avenanthramides, exclusive to oats, or their metabolites, have demonstrated anti‐cancer activity in cell and animal models^14^, ^15^, ^17^, ^18^ and inhibitory effects against human colon cancer cell proliferation.^19^ Potential mechanisms associated with ferulic acid, the predominant polyphenol in wheat,^8^ include transformation by intestinal microflora and probiotics to 4‐vinylguaiacol, with both compounds demonstrating in vitro anti‐proliferative effects against human CRC cell lines HT‐29 and HCT‐116.^20^ Lignan metabolites enterolactone and enterodiol also inhibited growth of human colonic cancer SW480 cells.^21^

What remains unclear is whether the protective effects against CRC observed with grain polyphenols in experimental models are seen in populations. There are inconsistent findings between case–control and prospective studies investigating polyphenols from the whole diet and CRC risk,^22^ and mixed effects reported with subclasses including flavonoids having inverse^23^ or no association^24^ with CRC. When stratified by gender, inconsistent associations with flavonoids^25^ and phenolic acid intake^26^ were found. In particular, there is a lack of data relating to intakes of cereal polyphenols and non‐flavonoid compounds, including phenolic acids, and CRC risk.^22^ Moreover, polyphenol data and CRC associations have not been explored in Australia; a country suffering high impacts from CRC.^1^

In this study we investigated the association between cereal‐derived polyphenols and CRC risk, in a large population‐based Australian cohort. We hypothesised that higher cereal polyphenol intakes would be associated with lower risk of colorectal cancer.

## 2 METHODS

### 2.1 Study population

**FIGURE 1:**
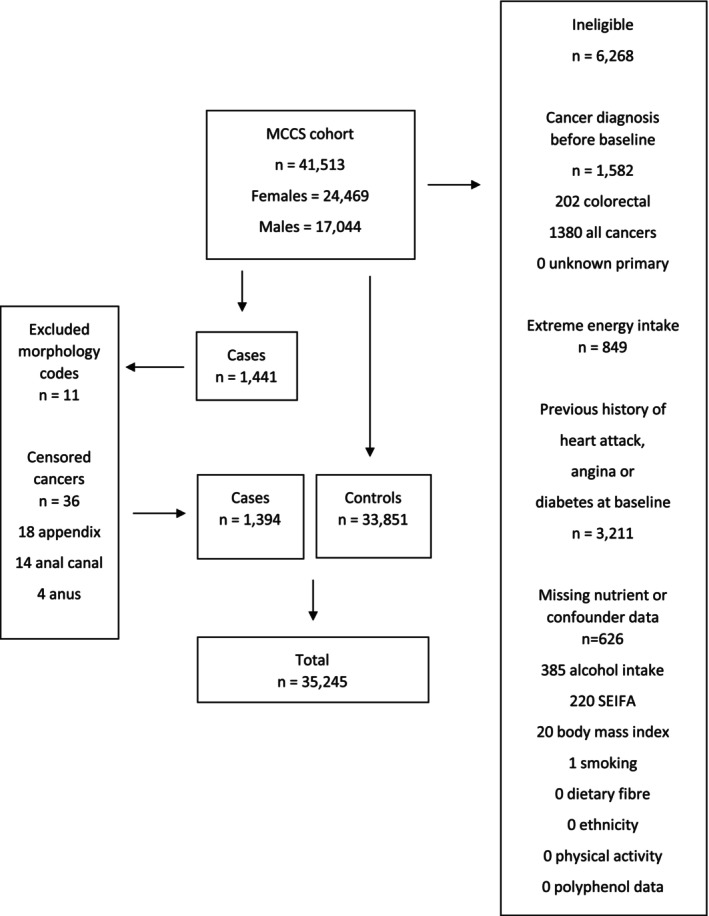
Flow diagram summarising inclusion criteria for MCCS participants for the survival analysis. MCCS, Melbourne Collaborative Cohort Study; SEIFA, Socio‐Economic Index for Areas.

The MCCS is a prospective cohort study comprising 41,513 adults aged 40–69 years, enrolled between 1990 and 1994 with baseline diet and lifestyle data collected to investigate diet–disease relationships. Most participants were Australian‐born (69%), with 13% born in Italy, 11% in Greece and 6% born in the United Kingdom.^2^ Further details of the MCCS are described elsewhere.^2^ Participants were ineligible for this study if they had pre‐baseline cancer diagnosis, or self‐reported pre‐baseline history of angina, heart attack or diabetes, as these may be associated with dietary alterations. Participants were also excluded if they had missing data on diet or for any confounder or reported extreme energy intake in the highest or lowest 1% of the sex‐specific distributions. This resulted in 6268 exclusions from a cohort of 41,513 participants, leaving a total of 35,245 participants eligible for analysis in the present study (Figure 1).

### 2.2 Polyphenol intake from cereal foods

#### 2.2.1 Diet data

Dietary intake of participants was measured at baseline using a self‐reported 121‐item food frequency questionnaire (FFQ) and ‘Eating Habits’ and ‘Lifestyle’ questionnaires that were designed for use in the MCCS.^27^ There are 17 items within the MCCS FFQ from the ‘Cereal foods, cakes and biscuits’ category, all included in the present study analyses, namely (1) wheatgerm, (2) muesli, (3) other breakfast cereals, (4) rice (boiled) including brown rice, (5) fried rice, (6) mixed dishes with rice, (7) white bread, rolls or toast, (8) wholewheat or rye bread, rolls or toast, (9) fruit bread, (10) crackers or crispbreads, (11) sweet biscuits, (12) cakes or sweet pastries, (13) puddings, (14) pasta or noodles, (15) pizza, (16) dim sims or spring rolls and (17) pies or savoury pastries. Quantities consumed were estimated by converting the intake frequencies of each food item to daily equivalents which were then multiplied by sex‐specific standard portions. Further details of the questionnaires are available in published literature.^2^, ^27^ The FFQ has been validated in studies with biomarkers of antioxidant intake^28^ and plasma phospholipid fatty acids.^28^

#### 2.2.2 Cereal polyphenol data

A study‐specific database of polyphenols from cereal food items was developed to match the 17 cereal foods items included in the MCCS 121‐item FFQ,^29^ including breakfast cereals, breads, rice dishes, pasta and sweet and savoury baked items (foods only, not supplements). The polyphenol classes and compounds assessed included lignans (lariciresinol, matairesinol, pinoresinol, secoisolariciresinol), alkylresorcinols (5‐*n*‐heneicosylresorcinol (C21:0), 5‐*n‐*heptadecylresorcinol (C17:0), 5‐*n*‐nonadecylresorcinol (C19:0), 5‐*n*‐pentacosylresorcinol (C25:0), 5‐*n*‐tricosylresorcinol (C23:0)) and phenolic acids (ferulic acid, avenanthramide 2c, avenanthramide 2p, avenanthramide 2f).

Classification of the polyphenols was based primarily as described in Phenol‐Explorer database (PED) version 3.6.^8^ FFQ items were originally matched to polyphenol data in PED,^8^ a comprehensive database of phenolic compounds that has been utilised in assessment of polyphenol intakes and colorectal cancer.^26^ Where data were unavailable, proxy items, or logical zeros, if it was likely that the item contained little or none of a particular polyphenol, were applied.^30^ The intention was to have no missing or blank data cells to minimise underestimation of polyphenol content.^31^ Missing and zero values were resolved by searching other literature for similar foods and polyphenol values.^32^

The FFQ cereal items were matched to polyphenol data mostly from published literature (Table S1) with PED^8^ used for the initial searches to match cereal foods with polyphenols and retention factors. Complex foods were deconstructed to their constituent components^30^ based on an Australian food composition database^33^ or Australian recipes available from the internet, or cookbooks. Retention factors were applied to cooking processes and compounds as relevant.^34^ The daily quantity of each food item consumed was multiplied by the polyphenol values in the MCCS cereal polyphenol database. Totals for each polyphenol were summed across all foods consumed for each individual compound.

#### 2.2.3 Exposure variable

The exposure variables were intakes of selected cereal polyphenols from polyphenol classes lignans, alkylresorcinols and phenolic acids. These were included as these polyphenol classes constituted the majority of polyphenols in grains and are associated with anti‐carcinogenic mechanisms in models of CRC.^35^, ^36^ Exposure was modelled as individual compounds, classes and total polyphenols. Quintiles based on cut‐points from the whole of the eligible cohort were used to categorise the exposure variables. There was little difference in distributions of intakes between males and females therefore quintiles were calculated for the whole cohort. The lowest quintile was used as the referent category. Intakes were not normally distributed; hence, medians and 25th and 75th percentiles are presented.

### 2.3 Medical, demographic and health data

Additional data collected at baseline include anthropometric measurements, and self‐reported data on smoking, alcohol consumption, physical activity, health conditions, details of medications taken and socioeconomic status.^2^ SES is defined as quintiles of Socio‐Economic Index for Areas ‐ Disadvantage (SEIFA): Q1 (most disadvantaged) to Q5 (least disadvantaged); physical activity was coded from least to most physically active (0, >0 to <4, 4 to <6, ≥6) to represent approximate quartiles of distribution, derived from frequency responses that were summed after assigning twice the weight to vigorous activity, where 0 (none), 1.5 (one or two times per week) and 4 (≥3 times per week) for walking, less vigorous and vigorous activity.

### 2.4 Cohort follow‐up and case ascertainment

Cases were identified from notifications to the Victorian Cancer Registry (VCR) and the AIHW Australian Cancer Database and deaths were ascertained through the Victorian Registry of Births, Deaths and Marriages and the National Death Index.

Incident colorectal cancers were defined (following the third Revision of the International Classification of Diseases for Oncology) as the primary invasive cancer of the colon (C18.0, C18.2–18.9) or rectum (C19.9, C20.9, C21.8). Tumours of the appendix (C18.1), anus (C21.0), anal canal (C21.1) were censored at date of diagnosis. The date 31 December 2017 was selected as end of follow‐up as complete cancer incidence data were available to that date.

### 2.5 Statistical analysis

Follow‐up commenced at study entry and ceased at the date of any of the following, whichever came first: date of death or date left Australia, at date of diagnosis of CRC, diagnosis of a tumour of the appendix (C18.1), anus (C21.0) or anal canal (C21.1) or date of diagnosis of unknown primary tumour. In the separate model for colon cancer, cancers of the rectum were censored at date of diagnosis and vice versa.

Potential confounders were identified from a directed acyclic graph (Figure S1) generated using DAGitty software v. 3.0^37^ and informed by associations shown with CRC in the literature. Thus, models were adjusted for socio‐economic status (SES), age, alcohol intake, dietary fibre intake, ethnicity, energy (kJ) intake, physical activity and smoking. Body mass index (BMI) was considered to be on the causal pathway thus included as a sensitivity analysis as baseline diet could potentially be determined by current BMI.

Multivariable Cox proportional hazards multivariable regression models with age as the time axis were used to estimate hazard ratios and 95% confidence intervals for the risk of CRC associated with polyphenol intake. The main model (Model 1) was adjusted for sex (female, male); SES (Q1 to Q5); alcohol consumption: never, ex‐drinker, low (<20 g/d), moderate (20–39 g/d), high intake (≥40 g/d); total fibre (g/d); country of birth (Australia/NZ/Other; UK/Malta, Italy, Greece); total energy intake (kJ/d); physical activity score (0, >0 to <4, 4 to <6, ≥6); smoking status (never, current, former). Model 2 was additionally adjusted for BMI as a sensitivity analysis. Additional sensitivity analyses were performed, excluding the first 2 years of follow‐up, and excluding participants using aspirin or non‐steroidal anti‐inflammatory drugs (NSAIDs), calcium supplements or fibre supplements at baseline.

The proportional hazards assumption was tested by Schoenfeld residuals and graphically. Statistical analysis was performed using Stata MP 16.1 statistical software.^38^ All statistical tests were two‐sided with *p* < 0.05 considered statistically significant.

This study is reported according to the Strengthening the Reporting of Observational Studies in Epidemiology—Nutritional Epidemiology (STROBE‐nut) framework (Table S2).^39^

## 3 RESULTS

The study includes an average of 22.4 years follow‐up, with 1394 incident cases included for analysis, comprising 676 males and 718 females. A total of 1441 individuals that met the case definition were initially identified; however, 11 were excluded due to irregular morphology codes (5 colon cancer cases, 5 rectal cancer cases, 1 overlapping site of rectum, anus and anal canal). Another 36 cases were censored that were not defined as cancers of the colon or rectum (18 appendix cancer (C18.1), 14 anal canal (C21.1) and 4 anus (C21.0)), leaving a total of 1394 colorectal cases (946 colon and 448 rectum) included for analysis.

### 3.1 Participant characteristic by quintiles of intake of polyphenols from cereal foods

Participant characteristics and dietary intakes of polyphenol classes are presented by quintiles of total cereal polyphenol intake (Table 1). The median (25th to 75th percentile) intake of total cereal polyphenols was 85.9 mg/d (50.9–150.8). Participants with higher intakes of total cereal polyphenols were more likely to be born in Australia/New Zealand, less disadvantaged, non‐smokers and to have higher physical activity scores. People in the highest quintile of intake were also more likely to be taking fibre and calcium supplements.

**TABLE 1:** Characteristics of eligible study participants in the MCCS at baseline (*n* = 35,245) by quintile^e^ of total cereal polyphenol intake.

Quintile lower and upper limits	Eligible *n* = 35,245	Q1 *n* = 7049 (2.32–42.74)	Q2 *n* = 7049 (42.75–72.98)	Q3 *n* = 7049 (72.99–102.60)	Q4 *n* = 7049 (102.61–178.61)	Q5 *n* = 7049 (178.62–808.26)
Demographics						
Female, *n* (%)	21,349 (61)	4183 (59)	4593 (65)	4259 (60)	4028 (57)	4286 (61)
Age, y, mean (SD)	54.7 (8.6)	54.1 (8.3)	53.9 (8.6)	54.1 (8.5)	55.3 (8.7)	56.0 (8.7)
Body mass index (kg/m^2^), mean (SD)	26.8 (4.4)	27.8 (4.5)	27.0 (4.4)	26.6 (4.3)	26.2 (4.1)	26.2 (4.2)
Country of birth, *n* (%)						
Australia/New Zealand	24,491 (69)	3536 (50)	4895 (69)	5075 (72)	5492 (78)	5493 (78)
United Kingdom/Malta	2607 (7)	400 (6)	496 (7)	558 (8)	565 (8)	588 (8)
Italy	4451 (13)	1581 (22)	1017 (14)	790 (11)	551 (8)	512 (7)
Greece	3696 (10)	1532 (22)	641 (9)	626 (9)	441 (6)	456 (7)
SEIFA, *n* (%)						
Q1	6194 (18)	1599 (23)	1368 (19)	1118 (16)	1047 (15)	1062 (15)
Q2	7301 (21)	1792 (25)	1559 (22)	1375 (20)	1260 (18)	1315 (19)
Q3	5561 (16)	1169 (17)	1147 (16)	1079 (15)	1051 (15)	1115 (16)
Q4	6546 (19)	1137 (16)	1222 (17)	1364 (19)	1386 (20)	1437 (20)
Q5	9643 (27)	1352 (19)	1753 (25)	2113 (30)	2305 (33)	2120 (30)
Lifestyle behaviours						
Smoking status, *n* (%)						
Never	20,796 (59)	3806 (54)	4149 (59)	4232 (60)	4274 (61)	4335 (62)
Former	10,547 (30)	1921 (27)	2040 (29)	2127 (30)	2233 (32)	2226 (32)
Current	3902 (11)	1322 (19)	860 (12)	690 (10)	542 (8)	488 (7)
Alcohol drinking status, *n* (%)						
Lifetime abstainers	9951 (28)	2316 (33)	2080 (30)	1808 (26)	1807 (26)	1940 (28)
Ex‐drinkers	3619 (10)	733 (10)	744 (11)	721 (10)	701 (10)	720 (10)
Low intake (<20 g/d)	14,960 (42)	2492 (35)	2940 (42)	3123 (44)	3235 (46)	3170 (45)
Medium intake (20–39 g/d)	4230 (12)	830 (12)	783 (11)	896 (13)	902 (13)	819 (12)
High intake (>40 g/d)	2485 (7)	678 (10)	502 (7)	501 (7)	404 (6)	400 (6)
Physical activity score, *n* (%)						
0	7748 (22)	2262 (32)	1660 (24)	1397 (20)	1216 (17)	1213 (17)
>0 to <4	7156 (20)	1496 (21)	1498 (21)	1437 (20)	1352 (19)	1373 (19)
4 to <6	12,263 (35)	2302 (33)	2423 (34)	2468 (35)	2546 (36)	2524 (36)
≥6	8078 (23)	989 (14)	1468 (21)	1747 (25)	1935 (27)	1939 (28)
Dietary intake						
Total cereal polyphenol intake (mg/d)^a^ ^,^ ^f^	85.9 (50.9–150.8)	26.2 (18.6–34.3)	58.7 (50.9–66.3)	85.9 (79.3–93.7)	126.7 (113.0–150.8)	224.0 (203.4–272.8)
Lignans (mg/d)^b^ ^,^ ^f^	0.49 (0.13–0.86)	0.08 (0.05–0.12)	0.20 (0.10–0.35)	0.50 (0.30–0.62)	0.72 (0.61–0.86)	1.72 (1.49–1.88)
Alkylresorcinols (mg/d)^c^ ^,^ ^f^	19.4 (10.7–34.1)	4.3 (2.8–6.5)	13.4 (10.6–16.0)	19.3 (16.5–21.8)	29.7 (26.2–34.2)	48.2 (42.1–60.5)
Phenolic acids (mg/d)^d^ ^,^ ^f^	66.3 (39.1–115.3)	21.4 (15.4–27.7)	44.7 (39.1–50.3)	66.3 (60.6–72.3)	97.1 (86.3–115.3)	174.0 (158.1–209.2)
Energy intake (kJ/d)^f^	8722 (6964‐10,897)	7431 (5815‐9328)	8049 (6451‐10,015)	8592 (7020‐10,626)	9196 (7562‐11,339)	10,286 (8579‐12,471)
Dietary fibre intake (g/d)^f^	29.1 (22.7–37.0)	21.0 (16.4–27.0)	25.1 (20.7–30.8)	28.5 (23.5–34.5)	32.7 (27.3–39.3)	38.7 (32.5–46.4)
Taking fibre supplements, *n* (%)						
Yes	6689 (19)	459 (7)	1037 (15)	1341 (19)	1915 (27)	1937 (27)
No	28,556 (81)	6590 (93)	6012 (85)	5708 (81)	5134 (73)	5112 (73)
Taking calcium supplements, *n* (%)						
Yes	3860 (11)	483 (7)	762 (11)	827 (12)	860 (12)	928 (13)
No	31,385 (89)	6566 (93)	6287 (89)	6222 (88)	6189 (88)	6121 (87)
Taking NSAIDs or aspirin, *n* (%)						
Yes	7580 (22)	1383 (20)	1521 (22)	1509 (21)	1613 (23)	1554 (22)
No	27,665 (78)	5666 (80)	5528 (78)	5540 (79)	5436 (77)	5495 (78)
Cases, *n* (%)	1394 (4)	311 (22)	245 (18)	282 (20)	253 (18)	303 (22)

Abbreviations: MCCS, Melbourne Collaborative Cohort Study. SD, standard deviation. SEIFA, Quintiles of Index of Relative Socio‐economic Disadvantage, Q1 (most disadvantaged) ‐ Q5 (least disadvantaged). NSAIDs, non‐steroidal anti‐inflammatory drugs.

^a^ Sum of alkylresorcinols, lignans and phenolic acids from cereal foods.

^b^ Sum of lariciresinol, matairesinol, pinoresinol and secoisolariciresinol from cereal foods.

^c^ Sum of 5‐*n*‐heneicosylresorcinol (C21:0), 5‐*n*‐heptadecylresorcinol (C17:0), 5‐*n*‐nonadecylresorcinol (C19:0), 5‐*n*‐pentacosylresorcinol (C25:0) and 5‐*n*‐tricosylresorcinol (C23:0) from cereal foods.

^d^ Sum of ferulic acid and avenanthramides (avenanthramide 2c, avenanthramide 2p and avenanthramide 2f) from cereal foods.

^e^ Percentages may not add up due to rounding.

^f^ Values presented as median (25th to 75th) percentile.

The intakes of all polyphenol classes, fibre and energy increased across quintiles of total cereal polyphenol intake. Phenolic acids were the most consumed compounds with a median intake of 66.3 mg/d (39.1–115.3) (Table 1). Alkylresorcinols contributed the second highest amount to total intakes, with a median of 19.4 mg/d (10.7–34.1) (Table 1). The least consumed cereal polyphenols were lignans with a median intake for the cohort of 0.49 mg/d (0.13–0.86) (Table 1). The results also show that there was a wide range of intakes of the measured polyphenol classes; for total polyphenols the median in the top quintiles was almost 10‐fold that of the lowest quintile (Table 1).

### 3.2 Colorectal cancer risk in relation to cereal polyphenol intake

Multivariable adjusted hazard ratios (HRs) and 95% confidence intervals (CIs) for polyphenol quintiles and CRC risk are presented (Table 2). There was no evidence of non‐proportional hazards. Results from the main model (without BMI) for total polyphenol intake showed a reduced risk of CRC associated with Q2 (HR: 0.80; 95% CI, 0.68–0.95) and Q4 (HR: 0.75; 95% CI, 0.62–0.90) compared with the lowest quintile. A similar pattern was also seen for phenolic acids. The association with alkylresorcinols showed a reduced risk of CRC with Q3 (HR: 0.80; 95% CI, 0.67–0.95) and Q4 (HR: 0.79; 95% CI, 0.66–0.95) compared with the lowest quintile, but not for Q5 (HR: 0.88; 95% CI, 0.73–1.07). The addition of BMI in Model 2 did not appreciably alter the results.

**TABLE 2:** Hazard ratios (HRs) and 95% confidence intervals (95% CIs) for colorectal cancer^a^ risk in relation to dietary intake of cereal polyphenols in the MCCS, by total intake and polyphenol class according to quintile of intake^b^.

			Model 1 without BMI^c^			Model 2 with BMI^c^		
			95% CI			95% CI		
	Quintiles	Number of cases/person years	HR			HR		
Polyphenols total^d^	1	311/156,691	1.00			1.00		
	2	245/158,994	0.80	0.68	0.95	0.81	0.68	0.96
	3	282/158,527	0.90	0.76	1.06	0.91	0.76	1.07
	4	253/157,497	0.75	0.62	0.90	0.77	0.64	0.92
	5	303/156,270	0.88	0.73	1.07	0.90	0.74	1.09
Lignans^e^	1	308/156,691	1.00			1.00		
	2	257/159,407	0.87	0.74	1.03	0.87	0.74	1.04
	3	269/158,977	0.95	0.80	1.13	0.96	0.81	1.14
	4	263/157,932	0.85	0.71	1.01	0.86	0.72	1.03
	5	297/156,368	0.94	0.78	1.14	0.96	0.79	1.15
Alkylresorcinols^f^	1	309/158,412	1.00			1.00		
	2	254/159,554	0.87	0.73	1.03	0.87	0.73	1.03
	3	262/158,235	0.80	0.67	0.95	0.81	0.68	0.96
	4	259/157,633	0.79	0.66	0.95	0.80	0.67	0.96
	5	310/155,541	0.88	0.73	1.07	0.89	0.74	1.08
Phenolic acids^g^	1	318/157,783	1.00			1.00		
	2	242/158,794	0.75	0.63	0.89	0.75	0.64	0.90
	3	279/158,831	0.88	0.74	1.04	0.89	0.75	1.05
	4	256/157,657	0.74	0.62	0.88	0.75	0.63	0.90
	5	299/156,310	0.84	0.70	1.02	0.86	0.71	1.04
Avenanthramides^h^	1	285/158,686	1.00			1.00		
	2	277/158,928	1.05	0.88	1.24	1.04	0.88	1.24
	3	275/159,493	1.03	0.87	1.23	1.04	0.87	1.24
	4	268/153,805	0.81	0.67	0.97	0.83	0.69	0.99
	5	289/158,463	1.04	0.86	1.25	1.06	0.89	1.28
Ferulic acid	1	317/157,815	1.00			1.00		
	2	242/158,747	0.75	0.63	0.89	0.76	0.64	0.90
	3	281/158,814	0.89	0.75	1.05	0.90	0.76	1.07
	4	254/157,674	0.74	0.61	0.88	0.75	0.62	0.90
	5	300/156,325	0.85	0.70	1.03	0.87	0.72	1.05

Abbreviations: BMI, body mass index; HR, hazard ratio; MCCS, Melbourne Collaborative Cohort Study; NSAIDs, non‐steroidal anti‐inflammatory drugs; SEIFA, Quintiles of Index of Relative Socio‐economic Disadvantage, Q1 (most disadvantaged)–Q5 (least disadvantaged).

^a^ Colon and rectal cancer cases combined.

^b^ Exposure is category into quintiles with the lowest quintile used as the referent category.

^c^ The association between cereal polyphenols and colorectal cancer was estimated by Cox proportional hazard models using age (years) as the time scale. Model 1 was adjusted for sex (female, male); SES defined as Socio‐Economic Index for Areas ‐ Disadvantage (SEIFA): Q1 (most disadvantaged) to Q5 (least disadvantaged); alcohol consumption: never, ex‐drinker, low (<20 g/d), moderate (20–39 g/d), high intake (≥ 40 g/d); total fibre (g/d); ethnicity, defined as country of birth (Australia/New Zealand; UK/Malta, Italy, Greece); total energy intake (kJ/d); physical activity score (0, >0 to <4, 4 to <6, ≥6); smoking status (never, current, former). Model 2 also included (BMI, continuous kg/m^2^).

^d^ Sum of alkylresorcinols, lignans and phenolic acids from cereal foods.

^e^ Sum of lariciresinol, matairesinol, pinoresinol and secoisolariciresinol from cereal foods.

^f^ Sum of 5‐*n*‐heneicosylresorcinol (C21:0), 5‐*n*‐heptadecylresorcinol (C17:0), 5‐*n*‐nonadecylresorcinol (C19:0), 5‐*n*‐pentacosylresorcinol (C25:0) and 5‐*n*‐tricosylresorcinol (C23:0) from cereal foods.

^g^ Sum of ferulic acid and avenanthramides (avenanthramide 2c, avenanthramide 2p and avenanthramide 2f) from cereal foods.

^h^ Sum of avenanthramides (avenanthramide 2c, avenanthramide 2p and avenanthramide 2f) from cereal foods.

The same associations were also investigated for colon and rectum cancers separately (Table 3). Results from the main model (without BMI) and Model 2 (with BMI) showed that there was no association for colon cancer, but the HRs were <1 for Q4 for total polyphenols and all subclasses other than lignans. There was no appreciable difference when BMI was added to the model. Reduced HRs observed in Q2 and Q4 with phenolic acids for colon cancer reflected the pattern for ferulic acid in both models, whereas for avenanthramides, reduced HR was evident only in Q4 for colon cancer. There was no association evident with cereal polyphenol intake and rectal cancer incidence, but the HRs were <1 for Q2 for phenolic acids that reflected the intake of ferulic acid.

**TABLE 3:** Hazard ratios (HRs) and 95% confidence intervals (95% CIs) for colon and rectal cancer in relation to dietary intake of cereal polyphenols, by total intake and polyphenol class according to quintile of intake^a^.

			Colon cancer Model 1^b^			Colon cancer Model 2^b^				Rectal cancer Model 1^b^			Rectal cancer Model 2^b^		
	Quintiles	Cases/person years	HR	95% CI		HR	95% CI		Cases/person years	HR	95% CI		HR	95% CI	
Polyphenols total^c^	1	201/158,088	1.00			1.00			110/158,088	1.00					
	2	176/158,994	0.85	0.69	1.04	0.85	0.69	1.05	69/158,994	0.71	0.52	0.96	0.71	0.52	0.97
	3	187/158,527	0.88	0.71	1.08	0.89	0.72	1.09	95/158,527	0.95	0.71	1.26	0.96	0.71	1.28
	4	168/157,497	0.72	0.57	0.90	0.73	0.58	0.92	85/157,497	0.83	0.60	1.13	0.84	0.61	1.16
	5	214/156,270	0.88	0.70	1.11	0.90	0.71	1.13	89/156,270	0.88	0.63	1.24	0.90	0.64	1.26
Lignans^d^	1	192/156,691	1.00			1.00			116/156,691	1.00					
	2	182/159,407	0.96	0.78	1.18	0.96	0.78	1.18	75/159,407	0.73	0.54	0.98	0.73	0.54	0.98
	3	189/158,977	1.05	0.85	1.29	1.05	0.85	1.30	80/158,977	0.80	0.59	1.08	0.80	0.59	1.08
	4	171/157,932	0.86	0.69	1.07	0.87	0.70	1.08	92/157,932	0.85	0.63	1.14	0.86	0.64	1.16
	5	212/156,368	1.02	0.81	1.28	1.03	0.82	1.29	85/156,368	0.81	0.59	1.13	0.83	0.60	1.15
Alkylresorcinols^e^	1	199/158,412	1.00			1.00			110/158,412	1.00			1.00		
	2	171/159,553	0.87	0.70	1.07	0.87	0.70	1.07	83/159,554	0.88	0.66	1.19	0.88	0.66	1.19
	3	185/158,235	0.84	0.68	1.03	0.84	0.68	1.04	77/158,235	0.73	0.54	0.99	0.74	0.54	1.00
	4	169/157,633	0.74	0.59	0.93	0.75	0.60	0.94	90/157,633	0.91	0.66	1.24	0.92	0.67	1.26
	5	222/155,541	0.90	0.71	1.14	0.91	0.72	1.15	88/155,541	0.83	0.59	1.17	0.85	0.60	1.19
Phenolic acids^f^	1	206/157,783	1.00			1.00			112/157,783	1.00			1.00		
	2	173/158,794	0.79	0.65	0.98	0.80	0.65	0.98	69/158,794	0.66	0.49	0.90	0.67	0.49	0.90
	3	185/158,831	0.86	0.70	1.06	0.87	0.71	1.07	94/158,831	0.92	0.69	1.24	0.93	0.70	1.25
	4	170/157,657	0.71	0.57	0.89	0.73	0.58	0.91	86/157,657	0.80	0.58	1.09	0.81	0.60	1.11
	5	212/156,310	0.85	0.68	1.07	0.87	0.69	1.09	87/156,310	0.83	0.59	1.16	0.84	0.60	1.18
Avenanthramides^g^	1	186/158,686	1.00			1.00			99/158,686	1.00			1.00		
	2	193/158,928	1.06	0.87	1.31	1.06	0.86	1.30	84/158,928	1.01	0.75	1.36	1.01	0.75	1.36
	3	186/159,493	0.98	0.79	1.21	0.99	0.80	1.22	89/159,493	1.15	0.85	1.56	1.15	0.85	1.57
	4	173/153,805	0.76	0.61	0.96	0.78	0.62	0.97	95/153,805	0.92	0.67	1.26	0.94	0.69	1.29
	5	208/158,463	1.06	0.85	1.32	1.09	0.87	1.36	81/158,463	0.98	0.70	1.36	1.01	0.72	1.40
Ferulic acid	1	205/157,815	1.00			1.00			112/157,815	1.00			1.00		
	2	174/158,747	0.80	0.65	0.99	0.81	0.66	0.99	68/158,747	0.65	0.48	0.88	0.65	0.48	0.89
	3	185/158,814	0.87	0.70	1.07	0.88	0.71	1.08	96/158,814	0.94	0.71	1.26	0.95	0.72	1.27
	4	169/157,674	0.71	0.57	0.89	0.73	0.58	0.91	85/157,674	0.79	0.58	1.08	0.80	0.59	1.10
	5	213/156,325	0.86	0.68	1.09	0.88	0.70	1.11	87/156,325	0.83	0.59	1.16	0.84	0.60	1.18

Abbreviations: BMI, body mass index; HR, hazard ratio; MCCS, Melbourne Collaborative Cohort Study; NSAIDs, non‐steroidal anti‐inflammatory drugs; SEIFA, Quintiles of Index of Relative Socio‐economic Disadvantage, Q1 (most disadvantaged)–Q5 (least disadvantaged).

^a^ Exposure is category into quintiles with the lowest quintile used as the referent category.

^b^ The association between cereal polyphenols and colorectal cancer was estimated by Cox proportional hazard models using age (years) as the time scale. Model 1 was adjusted for sex (female, male); SES defined as Socio‐Economic Index for Areas ‐ Disadvantage (SEIFA): Q1 (most disadvantaged) to Q5 (least disadvantaged); alcohol consumption: never, ex‐drinker, low (<20 g/d), moderate (20–39 g/d), high intake (≥ 40 g/d); total fibre (g/d); ethnicity, defined as country of birth (Australia/New Zealand; UK/Malta, Italy, Greece); total energy intake (kJ/d); physical activity score (0, >0 to <4, 4 to <6, ≥6); smoking status (never, current, former). Model 2 also included (BMI, continuous kg/m^2^).

^c^ Sum of alkylresorcinols, lignans and phenolic acids from cereal foods.

^d^ Sum of lariciresinol, matairesinol, pinoresinol and secoisolariciresinol from cereal foods.

^e^ Sum of 5‐*n*‐heneicosylresorcinol (C21:0), 5‐*n*‐heptadecylresorcinol (C17:0), 5‐*n*‐nonadecylresorcinol (C19:0), 5‐*n*‐pentacosylresorcinol (C25:0) and 5‐*n*‐tricosylresorcinol (C23:0) from cereal foods.

^f^ Sum of ferulic acid and avenanthramides (avenanthramide 2c, avenanthramide 2p and avenanthramide 2f) from cereal foods.

^g^ Sum of avenanthramides (avenanthramide 2c, avenanthramide 2p and avenanthramide 2f) from cereal foods.

Results of the sensitivity analyses excluding the first 2 years of follow‐up, and participants who used NSAIDs or aspirin, fibre or calcium supplements were similar to the results of the main analysis (Table S3).

## 4 DISCUSSION

CRC is a major cause of morbidity and mortality, and polyphenols within cereal foods may play a role in the protective associations of cereal foods in reducing CRC risk. Very little evidence of an association between CRC risk and intake of total cereal polyphenols or cereal polyphenol classes was observed in this study. The findings do not support our hypothesis that higher dietary intake of cereal polyphenols is associated with lower risk of CRC. There was no association for either colon or rectum cancer with total cereal polyphenols, but the HRs were <1 for Q4 and Q2, and the reduced HRs observed with phenolic acids and colon cancer were reflected in ferulic acid, but not avenanthramides.

Phenolic acids were the main compounds contributing to intakes of cereal polyphenols in the MCCS. Phenolic acids may have a role in reducing CRC risk as it has been demonstrated in human colon cancer cells that growth is inhibited by ferulic acid^20^ and avenanthramides.^40^ Phenolic acids, in particular, ferulic acid, are associated with grains such as wheat^41^ and wheat products were commonly consumed in the MCCS. Avenanthramides are phenolic acid compounds limited to oats;^42^ however, intake was small and likely due to limited consumption of oat‐based cereal foods (muesli and porridge) in this cohort. In the present study, there was no association between intake of phenolic acids from cereal foods, either as ferulic acid or avenanthramides and CRC risk. The European Prospective Investigation into Cancer and Nutrition (EPIC) study reported an inverse association for phenolic acids from all foods with colon cancer in men, and a positive association with rectal cancer in women.^26^ However, the association was for phenolic acids from the whole diet which was highly correlated with coffee intake.^26^ This was in contrast to the present study where the analysis was limited to cereal‐derived polyphenols. Coffee polyphenols are different to cereal polyphenols, though various types of phenolic acids are found in both.

Alkylresorcinols are phenolic lipid compounds that are derived mainly from wheat and rye,^43^ and in the MCCS represented the second highest contributors to cereal polyphenol intakes. Alkylresorcinols are reported to have experimental anti‐cancer properties including inhibiting colon cancer growth and inducing apoptosis.^15^ However, in our study there was no clear association between alkylresorcinol intake and CRC risk, intakes in Q3 and Q4 but not Q5 were associated with reduced HRs. In EPIC, no association was observed for alkylphenol intake and CRC.^26^ One EPIC analysis reported that the median (5th to 95th percentile) intake of alkylphenols (includes alkylresorcinols) for women was 24.4 mg/d (2.0–80.1) and men mg/d 39.7 (2.3–113.5)^26^ which is generally in agreement with our study [19.4 mg/d (10.7–34.1)].

Lignan phenolic compounds represented the smallest contributors to cereal polyphenol intakes in the MCCS. Potential anti‐carcinogenic effects of lignans include antioxidant activity, estrogenic and anti‐cancer activity; however, bioavailability of ingested dietary lignans is unclear.^44^ In the present study, there was no association between intakes of cereal‐derived lignans and CRC risk which may be due to the comparatively low intakes. Other studies measuring associations between dietary lignan intakes and CRC risk were from all foods, and not just cereals, and found either no association as in the EPIC study^26^ or an inverse association with colon cancer risk in the Bellvitge CRC case–control study.^23^

The association between baseline plasma polyphenol concentrations and CRC risk in other studies is unclear. In the EPIC study, there was an inverse association between colon cancer risk and equol, (an isoflavone metabolite), no association with plasma hydroxycinnamic acids, including ferulic acid^45^ but an increased risk of colon cancer found with homovanillic acid,^45^ a phenolic acid compound found in black and green olives, olive oil and beer.^46^ In a case–control study nested within the EPIC study, higher concentrations of plasma alkylresorcinols were not associated with overall CRC, proximal colon cancer or rectal cancer, but were associated with a lower incidence of distal colon cancer.^47^ In the Nordic health and whole‐grain consumption (HELGA) cohort, which was part of EPIC, the associations between CRC risk and both plasma alkylresorcinols and intake of wholegrain assessed by FFQ were examined in a nested case–control study.^48^ Similar to the EPIC analysis that used plasma concentrations to assess exposure,^47^ an inverse association between distal colon cancer incidence and plasma alkylresorcinol concentration was noted.^48^ However, there was no association for wholegrain intake,^48^ consistent with the present study and the EPIC study using dietary questionnaires to measure exposure.^26^

It is important to note that circulating concentrations of compounds are not necessarily reflective of dietary intake.^49^ The lack of association of cereal polyphenol intake with CRC in the present study may also be partly explained by the effects of the cereal food matrix. Wheat phenolic acids, such as ferulic acid, are usually found as insoluble compounds bound to cell wall components such as lignins.^41^ Free ferulic acid is reported to be well absorbed; however, when esterified, as in grains and cereals, absorption may be minimal compared with the soluble fraction.^49^ When ferulic acid was fed to rats in cereal diets compared with semi‐purified diets, the cereal matrix severely limited ferulic acid bioavailability.^50^

Estimating polyphenol intakes is challenged by the limitations of matching polyphenol datasets with food frequency data, and potentially a source of estimation error. FFQs are practical and efficient tools for measuring dietary intakes in prospective studies, but it is well recognised that intakes are measured with considerable error.^51^ We also recognise that polyphenol intake estimation routinely relies on extracting data from external polyphenol datasets, with variations in extraction and analysis methods,^52^ gaps in available retention factor data particularly for cereal foods^34^ and possible under‐reporting of non‐extractable polyphenols.^53^ Where available, the data used for this study reported values after hydrolysis, as hydrolysis is used to liberate compounds bound to the food matrix.^54^ Additional variation is recognised in the polyphenol concentration within foods, that may arise due to genetic variability, differences in degree of ripeness and environmental factors such as exposure to light, plant stress, food processing and storage time^55^ and wide variation in nutrients and bioactive compounds in similar foods even grown in the same region.^56^ Individual variability in digestion and absorption and diet quality are also factors that have not been accounted for in this study and future studies utilising biomarkers in conjunction with dietary intake using diet records may add more clarity to diet–disease relationships.

The strengths of the present study include the large cohort and its prospective design, which minimises recall bias as data are collected years before the event. The Cox proportional hazard model was able to assess several covariates simultaneously and sensitivity analyses provided clarification regarding the effects of potential reverse causality or additional confounding, supported by a directed acyclic graph. This study used a complete case analysis as the number of participants with missing data on dietary or confounder variables was assessed as relatively small and unlikely to have a significant effect on the final results.^57^, ^58^

The use of a cohort‐specific FFQ that included 17 cereal foods was also a strength of the study; however, it was not designed with the aim of capturing polyphenol data and relied on a baseline FFQ to estimate across the entire follow‐up period. To improve the accuracy of calculating polyphenol intakes, we utilised multiple polyphenol datasets to match the FFQ items with specific cereal data applicable to the cohort, applied proxy and logical zero values to avoid missing data, and focussed on key cereal polyphenols associated with the foods consumed in the cohort. While there may be some similarities with food composition in international datasets, and Australians consume a lot of imported foods, it is also not feasible to conduct a full analysis of cereal polyphenols in Australian foods and we appreciate that not all cereal‐related polyphenols were captured in our assessment. Likewise, we acknowledge that polyphenols are widely dispersed in the food supply and may have anti‐cancer effects,^59^ and while we considered confounders that we had available data for using an evidence‐informed directed acyclic graph, a body of polyphenols represented in other food groups could be additional confounders. However, it is reasonable to assume that a study participant consuming a high/low polyphenol intake will still have a relatively high/low intake, irrespective of which database or dataset is used.

The present study provides new information regarding the potential role of cereal polyphenols in CRC aetiology. The findings do not support our hypothesis that higher intakes of cereal polyphenols are associated with reduced CRC risk; however, the association with total polyphenols or other polyphenol classes is still unclear. The present findings reflect other prospective cohort studies that do not show the same protective effects of polyphenols from all foods when compared with some case–control studies. Cereal polyphenol intakes measured in the current study are in agreement with other studies for alkylresorcinols, but there was limited cohort data from other studies to compare with intakes of cereal derived lignans and phenolic acids reported in this study. The lack of association in this study between CRC risk and cereal polyphenols may be partly reflective of some of the limitations of the diet data and the indirect method of assessment of polyphenol values. Further studies are warranted to expand the polyphenol compounds and food groups analysed to add to the information derived from prospective cohorts about dietary factors in CRC aetiology. Direct assessment methods using biomarkers have been used in other studies^45^ and in future studies may provide further clarity if used in combination with dietary assessment.

## AUTHOR CONTRIBUTIONS

**Kristina Vingrys:** Conceptualization (equal); data curation (equal); formal analysis (equal); investigation (equal); methodology (equal); project administration (lead); writing – original draft (lead); writing – review and editing (lead). **Michael Mathai:** Conceptualization (supporting); formal analysis (supporting); supervision (supporting); writing – review and editing (supporting). **Andrew McAinch:** Conceptualization (equal); formal analysis (equal); investigation (supporting); methodology (equal); supervision (lead); writing – original draft (equal); writing – review and editing (equal). **Julie K. Bassett:** Conceptualization (supporting); data curation (equal); formal analysis (equal); methodology (equal); writing – review and editing (equal). **Maximilian deCourten:** Conceptualization (supporting); formal analysis (supporting); methodology (supporting); supervision (supporting); writing – review and editing (equal). **Lily Stojanovska:** Conceptualization (supporting); formal analysis (supporting); supervision (supporting); writing – review and editing (supporting). **Lynne Millar:** Conceptualization (supporting); formal analysis (supporting); methodology (supporting); writing – review and editing (equal). **Graham G Giles:** Data curation (lead); formal analysis (supporting); funding acquisition (lead); methodology (supporting); project administration (lead); resources (lead); writing – review and editing (supporting). **Allison Hodge:** Conceptualization (equal); data curation (lead); formal analysis (equal); investigation (equal); methodology (equal); project administration (equal); resources (equal); supervision (lead); writing – original draft (equal); writing – review and editing (equal). **Vasso Apostolopoulos:** Conceptualization (equal); formal analysis (supporting); methodology (supporting); supervision (lead); writing – original draft (equal); writing – review and editing (equal).

## FUNDING INFORMATION

Melbourne Collaborative Cohort Study (MCCS) cohort recruitment was funded by VicHealth and Cancer Council Victoria. The MCCS was further augmented by Australian National Health and Medical Research Council grants 209057, 396414 and 1074383 and by infrastructure provided by Cancer Council Victoria. The first author was also supported by Victoria University Postgraduate Research Scholarship and infrastructure provided by Victoria University.

The funding bodies had no role in the design, analysis or writing of this article.

## CONFLICT OF INTEREST STATEMENT

The authors have no conflict of interest to disclose.

## ETHICAL APPROVAL STATEMENT

This study was conducted according to the guidelines laid down in the Declaration of Helsinki and all procedures involving research study participants were approved by the Cancer Council Victoria Human Research Ethics Committee (IEC9001) and Victoria University Human Research Ethics Committee (HRE 16‐289). Written informed consent was obtained from all participants.

## Supporting information


Figure S1.
Table S1.
Table S2.
Table S3.

## Data Availability

Enquiries regarding data access should be directed in the first instance to the corresponding author. Further details about using MCCS data can be found at the data administrators' website: https://www.pedigree.org.au/default.aspx.
