# Supplementary material for: Intake of polyphenols from cereal foods and colorectal cancer risk in the Melbourne Collaborative Cohort Study
Source: Cancer Med. 2023 Sep 13;12(18):19188–202. doi: 10.1002/cam4.6514 (PMC10557875; doi:10.1002/cam4.6514)
Supplement: Supplementary file 1 — Figure S1. Table S1. Table S2. Table S3. [file CAM4-12-19188-s001.docx]

**Supplementary Table S1.** Published literature used to create the polyphenol database for calculating dietary intakes of cereal polyphenols for the Melbourne Collaborative Cohort Study.

| **Polyphenol class** | **Individual polyphenols^a^** | **Database origin and reference** |
| --- | --- | --- |
| Alkylresorcinols | 5-*n*-heneicosylresorcinol (C21:0), 5-*n-*heptadecylresorcinol (C17:0), 5-*n*-nonadecylresorcinol (C19:0), 5-*n*-pentacosylresorcinol (C25:0), 5-*n*-tricosylresorcinol (C23:0) | Finland ^1^, Sweden ^2-4^ |
| Lignans | Lariciresinol, matairesinol, pinoresinol, secoisolariciresinol | Canada ^5^, The Netherlands ^6^, UK ^7 b^ ; US, Finland, UK ^8 b^ |
| Phenolic acids | Avenanthramide 2p, avenanthramide 2f , avenanthramide 2c  Ferulic acid | European ^9^; Finland ^1^; Sweden, UK, US ^10^; The UK ^11^  Finland ^1^ |

UK = United Kingdom; US = United States

^a^ Compound descriptions may vary slightly in publications and databases

^b^ Matairesinol and secoisolariciresinol only

**Supplementary Table S3.** Sensitivity analyses excluding the first 2-years of follow-up, NSAID^a^ use (NSAIDs or aspirin), fibre supplement use (wheat bran fibre: yes/no; oat bran fibre: yes/no; and fibre supplement: yes/no) and calcium supplement use (yes/no).

| Polyphenol class |  | Exclude first 2 years of follow-up* | | | Exclude NSAIDs users** | | | Exclude fibre supplement users* | | | | Exclude calcium supplement users* | | |
| --- | --- | --- | --- | --- | --- | --- | --- | --- | --- | --- | --- | --- | --- | --- |
|  | Quintiles | HR | Lower bound | Upper  bound | HR | Lower bound | Upper bound | | HR | Lower bound | Upper  bound | HR | Lower bound | Upper  bound |
| Polyphenols total |  |  |  |  |  |  |  | |  |  |  |  |  |  |
|  | 2 | 0.78 | 0.66 | 0.93 | 0.76 | 0.63 | 0.92 | | 0.83 | 0.70 | 1.00 | 0.77 | 0.64 | 0.92 |
|  | 3 | 0.87 | 0.73 | 1.04 | 0.86 | 0.71 | 1.03 | | 0.91 | 0.76 | 1.09 | 0.87 | 0.73 | 1.04 |
|  | 4 | 0.73 | 0.60 | 0.88 | 0.67 | 0.55 | 0.83 | | 0.71 | 0.58 | 0.87 | 0.73 | 0.60 | 0.89 |
|  | 5 | 0.87 | 0.71 | 1.06 | 0.83 | 0.67 | 1.02 | | 0.85 | 0.68 | 1.05 | 0.86 | 0.70 | 1.05 |
| Lignans |  |  |  |  |  |  |  | |  |  |  |  |  |  |
|  | 2 | 0.89 | 0.75 | 1.06 | 0.85 | 0.70 | 1.02 | | 0.91 | 0.76 | 1.09 | 0.88 | 0.74 | 1.05 |
|  | 3 | 0.96 | 0.81 | 1.15 | 0.91 | 0.75 | 1.10 | | 0.93 | 0.77 | 1.13 | 0.93 | 0.77 | 1.11 |
|  | 4 | 0.84 | 0.70 | 1.01 | 0.77 | 0.63 | 0.94 | | 0.80 | 0.66 | 0.98 | 0.84 | 0.70 | 1.01 |
|  | 5 | 0.95 | 0.78 | 1.15 | 0.88 | 0.71 | 1.08 | | 0.88 | 0.71 | 1.08 | 0.94 | 0.77 | 1.14 |
| Alkylresorcinols |  |  |  |  |  |  |  | |  |  |  |  |  |  |
|  | 2 | 0.85 | 0.72 | 1.02 | 0.79 | 0.65 | 0.95 | | 0.87 | 0.73 | 1.05 | 0.82 | 0.68 | 0.98 |
|  | 3 | 0.80 | 0.67 | 0.95 | 0.73 | 0.60 | 0.89 | | 0.83 | 0.69 | 1.00 | 0.78 | 0.65 | 0.94 |
|  | 4 | 0.75 | 0.62 | 0.91 | 0.70 | 0.57 | 0.86 | | 0.72 | 0.59 | 0.88 | 0.77 | 0.63 | 0.93 |
|  | 5 | 0.87 | 0.72 | 1.06 | 0.81 | 0.65 | 0.99 | | 0.85 | 0.69 | 1.05 | 0.85 | 0.70 | 1.04 |
| Phenolic acids |  |  |  |  |  |  |  | |  |  |  |  |  |  |
|  | 2 | 0.73 | 0.61 | 0.87 | 0.71 | 0.59 | 0.85 | | 0.76 | 0.63 | 0.91 | 0.70 | 0.59 | 0.84 |
|  | 3 | 0.86 | 0.73 | 1.03 | 0.82 | 0.68 | 0.99 | | 0.89 | 0.75 | 1.07 | 0.86 | 0.72 | 1.02 |
|  | 4 | 0.71 | 0.59 | 0.86 | 0.67 | 0.55 | 0.82 | | 0.70 | 0.57 | 0.86 | 0.70 | 0.58 | 0.85 |
|  | 5 | 0.84 | 0.69 | 1.02 | 0.79 | 0.64 | 0.97 | | 0.80 | 0.65 | 0.99 | 0.82 | 0.67 | 1.00 |
| Avenanthramides |  |  |  |  |  |  |  | |  |  |  |  |  |  |
|  | 2 | 1.01 | 0.85 | 1.20 | 1.04 | 0.86 | 1.25 | | 1.06 | 0.89 | 1.26 | 1.02 | 0.86 | 1.22 |
|  | 3 | 1.00 | 0.84 | 1.20 | 1.03 | 0.85 | 1.25 | | 1.03 | 0.85 | 1.25 | 0.99 | 0.82 | 1.19 |
|  | 4 | 0.79 | 0.65 | 0.95 | 0.78 | 0.64 | 0.96 | | 0.85 | 0.70 | 1.04 | 0.80 | 0.66 | 0.97 |
|  | 5 | 1.02 | 0.85 | 1.23 | 1.03 | 0.84 | 1.26 | | 1.00 | 0.81 | 1.23 | 1.00 | 0.82 | 1.21 |
| Ferulic acid | 1 | 1.00 |  |  | 1.00 |  |  | | 1.00 |  |  | 1.00 |  |  |
|  | 2 | 0.73 | 0.61 | 0.87 | 0.71 | 0.59 | 0.86 | | 0.76 | 0.63 | 0.91 | 0.71 | 0.59 | 0.85 |
|  | 3 | 0.87 | 0.73 | 1.03 | 0.83 | 0.69 | 1.00 | | 0.91 | 0.76 | 1.09 | 0.86 | 0.72 | 1.03 |
|  | 4 | 0.71 | 0.59 | 0.85 | 0.66 | 0.54 | 0.81 | | 0.70 | 0.57 | 0.86 | 0.70 | 0.58 | 0.85 |
|  | 5 | 0.84 | 0.69 | 1.02 | 0.80 | 0.65 | 0.98 | | 0.80 | 0.65 | 0.99 | 0.83 | 0.68 | 1.01 |

^a^ Non-steroidal anti-inflammatory drug.

*Model adjusted for age, sex, alcohol intake, ethnicity, physical activity, smoking, SEIFA, dietary energy intake and fibre intake.

**Model adjusted for age, sex, alcohol intake, ethnicity, physical activity, smoking, dietary energy intake and fibre intake, and stratified for SEIFA.


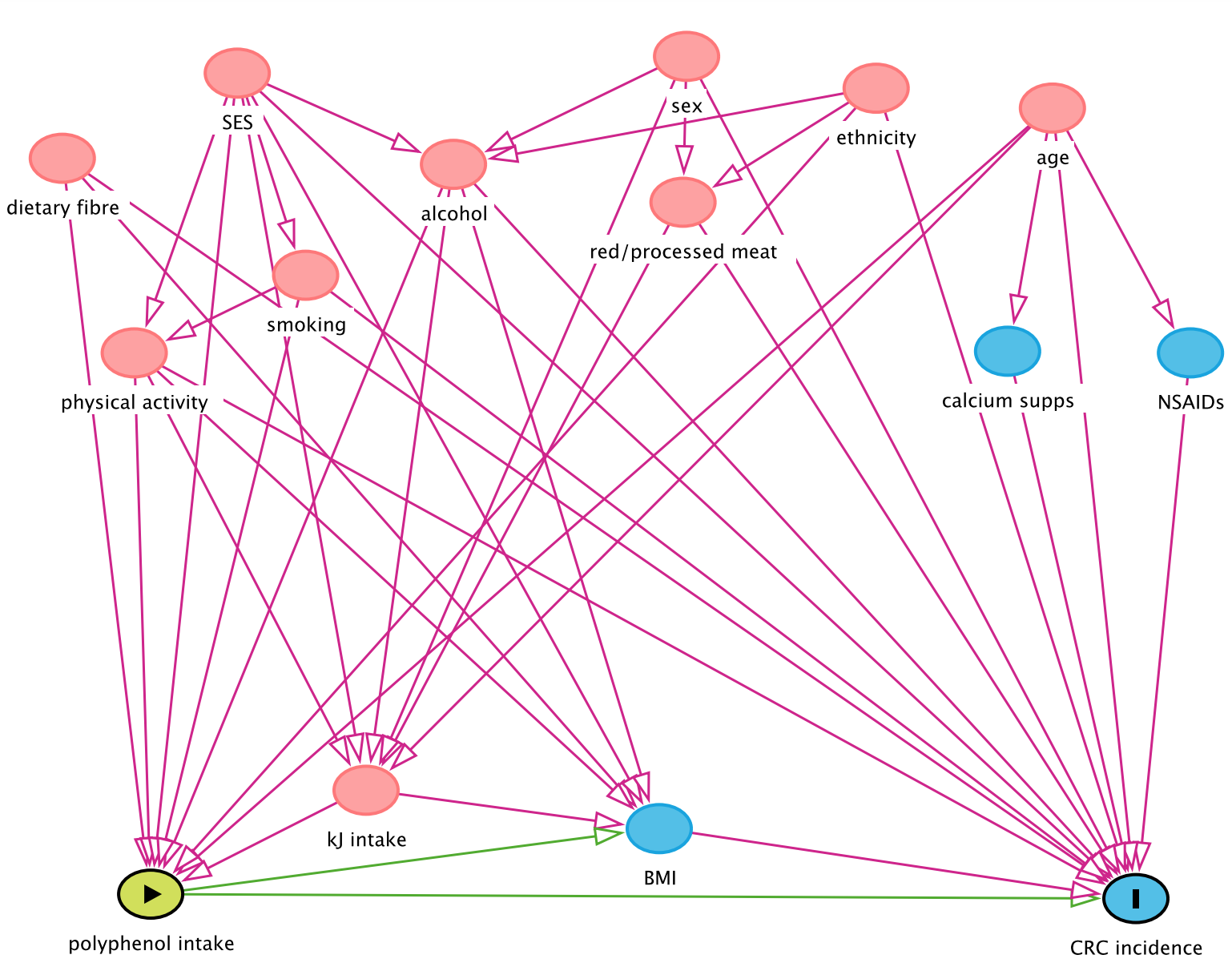


**Supplementary Figure S1.** Directed Acyclic Graph (DAG) used to identify covariates in the survival models to minimise confounding bias when estimating the association between exposure (dietary polyphenol intake) and outcome (CRC incidence).

Body mass index (BMI) is shown as an intermediate / mediator between the exposure and the outcome (exposure > BMI > outcome). Covariates were identified using the literature and matched to available MCCS data. Variables that were predictors of at least two other covariates were included ^12^. Red circles represent confounders, being ancestors of both exposure and outcome, whereas blue circles represent ancestors of the outcome only. Red lines reflect biasing paths and green lines reflect causal paths. Minimal sufficient adjustment sets for estimating the total effect of polyphenol intake (exposure) on colorectal cancer (CRC) incidence (outcome): SES, age, alcohol, dietary fibre, ethnicity, kJ intake, physical activity and smoking. The DAG was drawn and interpreted using DAGitty software ^13^. BMI: body mass index. CRC: colorectal cancer. kJ: kilojoule. MCCS: Melbourne Collaborative Cohort Study. NSAIDs: non-steroidal anti-inflammatory drugs. SES: socioeconomic status.

**References**

1. Mattila P, Pihlava J-m, Hellström J. Contents of phenolic acids, alkyl- and alkenylresorcinols, and avenanthramides in commercial grain products. *Journal of Agricultural and Food Chemistry*. 2005/10/01 2005;53(21):8290-8295. doi:10.1021/jf051437z

2. Ross AB, Shepherd MJ, Schüpphaus M, Sinclair V, Alfaro B, Kamal-Eldin A, Aman P. Alkylresorcinols in cereals and cereal products. *Journal of Agricultural and Food Chemistry* Jul 2 2003;51(14):4111-8. doi:10.1021/jf0340456

3. Chen Y, Ross AB, Aman P, Kamal-Eldin A. Alkylresorcinols as markers of whole grain wheat and rye in cereal products. *Journal of Agricultural and Food chemistry*. Dec 29 2004;52(26):8242-6. doi:10.1021/jf049726v

4. Landberg R, Kamal-Eldin A, Andersson A, Vessby B, Åman P. Alkylresorcinols as biomarkers of whole-grain wheat and rye intake: plasma concentration and intake estimated from dietary records. *Am J Clin Nutr*. 2008;87(4):832-838. doi:10.1093/ajcn/87.4.832

5. Thompson LU, Boucher BA, Liu Z, Cotterchio M, Kreiger N. Phytoestrogen content of foods consumed in Canada, including isoflavones, lignans, and coumestan. *Nutrition & Cancer*. 2006;54(2):184-201.

6. Milder IEJ, Arts ICW, Putte Bvd, Venema DP, Hollman PCH. Lignan contents of Dutch plant foods: a database including lariciresinol, pinoresinol, secoisolariciresinol and matairesinol. *British Journal of Nutrition*. 2005;93(3):393-402. doi:10.1079/BJN20051371

7. Kuhnle GGC, Dell'Aquila C, Aspinall SM, Runswick SA, Mulligan AA, Bingham SA. Phytoestrogen content of cereals and cereal-based foods consumed in the UK. *Nutr Cancer*. 01/01/ 2009;61(3):302-309. doi:10.1080/01635580802567141

8. Mazur W, Fotsis T, Wähälä K, Ojala S, Salakka A, Adlercreutz H. Isotope dilution gas chromatographic–mass spectrometric method for the determination of isoflavonoids, coumestrol, and lignans in food samples. *Anal Biochem*. 1996/01/15/ 1996;233(2):169-180. doi:https://doi.org/10.1006/abio.1996.0025

9. Shewry PR, Piironen V, Lampi A-M, Nyström L, Li L, Rakszegi M, Fraś A, Boros D, Gebruers K, Courtin CM, Delcour JA, Andersson AAM, Dimberg L, Bedő Z, Ward JL. Phytochemical and fiber components in oat varieties in the HEALTHGRAIN diversity screen. *Journal of Agricultural and Food Chemistry*. 2008/11/12 2008;56(21):9777-9784. doi:10.1021/jf801880d

10. Pridal AA, Böttger W, Ross AB. Analysis of avenanthramides in oat products and estimation of avenanthramide intake in humans. *Food Chem*. Jul 1 2018;253:93-100. doi:10.1016/j.foodchem.2018.01.138

11. Soycan G, Schär MY, Kristek A, Boberska J, Alsharif SNS, Corona G, Shewry PR, Spencer JPE. Composition and content of phenolic acids and avenanthramides in commercial oat products: Are oats an important polyphenol source for consumers? *Food Chemistry: X*. 2019/09/30/ 2019;3:100047. doi:https://doi.org/10.1016/j.fochx.2019.100047

12. Ferguson KD, McCann M, Katikireddi SV, Thomson H, Green MJ, Smith DJ, Lewsey JD. Evidence synthesis for constructing directed acyclic graphs (ESC-DAGs): a novel and systematic method for building directed acyclic graphs. *Int J Epidemiol*. 2020;49(1):322-329. doi:10.1093/ije/dyz150

13. Textor J, Hardt J, Knuppel S. DAGitty: a graphical tool for analyzing causal diagrams. Letter. *Epidemiology*. Sep 2011;22(5):745. doi:10.1097/EDE.0b013e318225c2be
